# The pain of a heart being broken: pain experience and use of analgesics by caregivers of patients with Alzheimer’s disease

**DOI:** 10.1186/s12888-015-0571-1

**Published:** 2015-07-28

**Authors:** Ewa Wojtyna, Katarzyna Popiołek

**Affiliations:** Institute of Psychology, University of Silesia, Katowice, ul. Grażyńskiego 53, 40-126 Katowice, Poland; Faculty in Katowice, University of Social Sciences and Humanities, ul. Techników 9, 40-326 Katowice, Poland

**Keywords:** Pain, Caregiver, Alzheimer’s disease, Social pain, Analgesic

## Abstract

**Background:**

It has been observed that psychical suffering (e.g. the feeling of losing a significant person) tends to reduce the physical pain tolerance threshold, as well as to increase the subjective sense of painfulness. The purpose of this study was to assess pain sensation among a group of caregivers of patients with Alzheimer’s disease, and to determine the psychological factors (emotional and relational) that contribute to both pain perception and coping with pain via the use of analgesics.

**Methods:**

The study comprised 127 caregivers of patients with Alzheimer’s disease. Questionnaires were used to elicit pain intensity, strength of emotional relationship between caregiver and patient, sense of painfulness of the loss experienced, depression level, and somatic ailments.

**Results:**

A large majority (87.4 %) of participants reported pain complaints, while 93 % took analgesics without a doctor’s recommendation at least once a week; 8 % took painkillers daily. The strongest predictors of both perceived pain and tendency to use analgesics were sense of loss and painfulness of loss in relation to the patient’s deteriorating condition.

**Conclusions:**

The pain experienced by caregivers may be connected to social pain resulting from the experience of losing someone they are close to. Caregivers may resort to excessive use of analgesics as a pain-coping strategy.

**Electronic supplementary material:**

The online version of this article (doi:10.1186/s12888-015-0571-1) contains supplementary material, which is available to authorized users.

## Background

Alzheimer’s disease (AD) is the most frequently occurring type of dementia. It is forecasted that in 2050 in Europe alone there will be 14.5 million people suffering from the disease [1]. Dysmnesia may be the most distinctive symptom of AD; yet for those who interact with the patient, the most troublesome symptoms are often behavior disorders, which accompany the deterioration of cognitive functions [2, 3]. Patients begin to behave differently than they used to: often they are aggressive, impulsive, sexually disinhibited or neglectful of their personal hygiene. A grave problem is that they endanger their own lives, as well as the lives of other people, e.g. by turning on gas or water, putting on clothes that are unsuitable for the weather, smoking near flammable objects, etc. Other problems that are extremely burdensome for caregivers are disturbances of circadian rhythm as well as patients’ tendency to wander about, sometimes away from their residences, which demands caregivers’ vigilance and application of special safety precautions. Patients may also be indiscriminate in their behavior. Another difficult and potentially painful symptom is that patients may not recognize their close family members, including caregivers themselves.

The burden of giving both physical and mental care to the AD patient results in frequent occurrence of symptoms of depression [4, 5], chronic fatigue, exhaustion, and sorrow [6] among caregivers. Our research to date has demonstrated that an important predictor of depression and exhaustion among people performing as caregivers is the type of bond they have with the person for whom they provide care: the stronger the relationship, the greater the emotional exhaustion of the caregiver [6]. Moreover, more profound feelings of loss connected with the deteriorating psychophysical condition of the patient and changes in her or his personality are associated with caregivers’ intensified symptoms of depression and diminished sense of personal achievement related to providing the care. It is interesting to note that a more profound sense of loss, coupled with strong positive emotional bonds and positive experiences with the patient when she or he was still healthy, has been associated with intensified somatic, rather than affective, symptoms of depression [7].

The results described are in alignment with the concept of social pain [8, 9]. In the present context, social pain is defined as a sensation of pain that arises not due to any tissue lesion, but as a result of a disturbance in substantial interpersonal relations. Frustration connected with the loss of a significant person and feelings of injustice or being treated unfairly result in activation of the anterior cingulate cortex, the same central nervous system area that is activated in the process of perceiving physical pain and that is connected with its affective dimension. It has been observed that social pain tends to reduce the physical pain tolerance threshold, as well as to increase the subjective sense of painfulness [9]. It is therefore reasonable to expect that the sense of losing a significant person as a consequence of AD-induced deterioration of the individual’s psychophysical condition is connected with intensification of pain sensations and/or a decreased capacity to cope with pain.

Given the current research context that associates a more frequent occurrence of somatic symptoms of depression in caregivers who have had positive experiences with the patient and who assess their bond with the patient as very positive and close, we hypothesized that pain sensations of these caregivers would be relatively intensified and the use of analgesics more frequent.

Our focus upon pain perception by caregivers looking after AD patients requires introduction of some specific terminology we use to describe that experience. Pain is a multi-dimensional phenomenon, and – in accordance with the definition provided by the International Association for the Study of Pain – an unpleasant sensory and emotional experience associated with actual or potential tissue damage, or described in terms of such damage [10]. This definition assumes the possibility that a pain sensation arises via both nociceptive and non-nociceptive mechanisms. In the latter case, pain may have its origins not only in damage suffered by nerve fibers (neuropathic pain), but also in psychogenic background.

The psychic dimension of pain is associated with attachment of an emotionally negative character to pain sensations. Pain is perceived as unpleasant and is connected with fear/anxiety or depression. This response is a substantial constituent of suffering, understood as a construct joining the physical, psychic, and existential dimensions of pain [11]. The affective dimension of pain is determined by limbic system structures – the same system referred to earlier as the neuropsychological basis of the social pain mechanism. The generation of social pain requires awareness of a threat to vital interpersonal relations and one’s own position as a participant in these relations. The notion of ‘sense of loss’ is used to connote the caregiver’s cognitive assessment of differences between the functioning of the AD patient now versus before the disease manifested. We assume that observations of that change create the basis for triggering the social pain mechanism. We therefore categorize the caregiver’s assessment of the patient’s deteriorating condition as the ‘sense of loss,’ and the suffering connected to that assessment as the ‘painfulness of loss.’

The aims of the study were to assess pain sensation among a group of caregivers of AD patients, and to determine the psychological factors (emotional and relational) that contribute to both pain perception and coping with pain by use of analgesics.

## Methods

The design was cross-sectional. Caregivers obtained data in the form of medical documentation on the severity of dementia symptoms from doctors. The remaining data were collected from caregivers via questionnaire. Participation by caregivers was voluntary, and participants were informed about the opportunity to receive treatment from a psychologist or psychotherapist if they felt that this would be helpful to them. Written informed consent for participation in the study was obtained from all participants. Ethics approval for the study was provided by the Ethics Committee of the University of Silesia.

### Participants

The sample comprised 127 primary caregivers of patients with AD. The participants were recruited from caregivers who accompanied the persons entrusted to their care for consultation in psycho-geriatric, geriatric, or psychiatric outpatient clinics, as well as in day care centers for patients with AD in several provinces of Poland. The criteria for inclusion in the study were: at least 1 year of providing care, at least 6 h of care every day, and direct contact with the patient, who had at least a moderate level of dementia. Exclusion criteria were: caregiver experiencing serious emotional disturbances and caregiver being a minor. The study was conducted between July and December 2013. Of all the caregivers who met the inclusion criteria (*n* = 358), 35.5 % agreed to complete the questionnaire. Those who declined to participate cited lack of time (94.4 %) or lack of interest (5.6 %). All those who declined and cited lack of time as their reason were also the sole caregiver of an individual with AD. The sociodemographic and care-related characteristics of participants are provided in Table 1.

**Table 1:** Characteristics of participants

Characteristics	N (%)	M	SD	Range
Gender				
Female	73 (57.5)			
Male	54 (42.5)			
Age [years]		53.21	21.84	31–83
Marital status				
Married	91 (71.7)			
Widowed	6 (4.7)			
Single	11 (8.7)			
Divorced	19 (14.9)			
Level of education				
Primary	9 (7.1)			
Technical/vocational	32 (25.2)			
Secondary	29 (22.8)			
University	57 (44.9)			
Employment status				
Working	76 (59.8)			
Living with patients	116 (91.3)			
Sole caregiver	98 (77.1)			
Time spent caring [h/day]		10.34	6.87	6–19
Time since onset of AD [months]		26.57	8.42	12–37
Pain				
Occurrence of pain-related somatic diseases	28 (22.0)			
Spondylosis	23 (18.1)			
Rheumatoid arthritis	4 (3.1)			
Phantom limb pain	1 (0.8)			
Pain level		4.13	4.28	0–10
Taking analgesics without a doctor’s recommendation [number of doses/week]		3.24	1.99	0–9
Strength of relationship with the patient				
Strength of emotional relationship		8.02	0.67	4–10
Positive experiences		7.21	1.99	0–10
Sense of loss		7.13	1.64	1–10
‘Painfulness’ of loss		6.45	2.12	1–10
Depression score		5.58	3.65	0–19

Note: *n* = 127

### Tools

#### Thermometer of Caregiver-Patient Strength of Emotional Relation

To characterize the quality of the emotional bond between the caregiver and the AD patient, the Thermometer of Caregiver-Patient Strength of Emotional Relation [6] developed by the authors was used. The tool consists of three questions, to which the participant responds by marking her/his choice on a visual-analog scale of 0 to 10: 1) ‘How strong is your emotional bond with the patient?’ (0 = ‘no emotional bond’; 10 = ‘extremely strong emotional bond’); 2) ‘What are your experiences with the patient from the pre-onset time?’ (0 = ‘extremely negative’; 10 = ‘extremely positive’); and 3) ‘Do you feel a sense of loss associated with the deteriorating condition of the patient?’ (0 = ‘no sense of loss’; 10 = ‘extremely severe sense of loss’). The results from these three scales were subsequently coded and introduced to statistical analyses as: ‘Strength of emotional relationship’, ‘Positive experiences’, and ‘Sense of loss’.

### Painfulness of Loss

The sense of painfulness of loss experienced by the caregiver in relation to the person she or he cares for was assessed in a similar way to the strength of emotional relationship between the caregiver and the patient, using the 0 to 10 single-item visual-analog scale, where 0 indicated no or minimum painfulness of loss experienced, and 10 signified maximum intensity of that feeling.

### Depression

To assess the level of depressive symptoms in caregivers, the Polish version of depression scale from the Hospital Anxiety and Depression Scale (HADS-D) was used [12, 13]. Participants express their agreement with each of seven statements, using a 4-point scale wherein higher scores represent greater levels of depressive affect, and a total score above 8 indicates a risk of clinical depression [12, 13]. Cronbach’s alpha for the HADS-D rendered a reliability level of 0.87 in our study.

### Somatic Ailments Questionnaire

This tool was developed by the authors to collect data on somatic diseases from which the caregiver was suffering, and drugs taken (Additional file 1). Separate questions concerned the frequency of taking analgesics, including those that do not require a prescription and are taken independently, without a doctor’s recommendation.

Among the somatic ailments, pain was distinguished. Participants were asked to state the level of pain they had experienced, on average, during the previous week, using the Numerical Rating Scale (from 0 = no pain, to 10 = maximum pain).

### Statistical analysis

The STATISTICA 10 package was used for analysis. Descriptive data are presented as means and standard deviations for continuous data and as frequencies and percentages for categorical data.

Kendall’s *tau-b* correlation coefficients were computed to examine the association of the three key participant variables (strength of the caregiver-patient emotional bond, sense of painfulness of loss connected with the deterioration of the patient’s condition, and intensification of depression) with the outcome variables of the intensity of pain perceived and frequency of taking analgesics without a doctor’s recommendation.

Logistic regression was used to model the probability of taking analgesics as a function of caregivers’ psychophysical condition and the caregiver–patient emotional bond. The dependent variable was established as daily or almost daily (4 to 7 days a week) use of analgesics by the caregiver, without a doctor’s recommendation. Predictor variables included a strong caregiver–patient relationship and substantial ‘painfulness’ of loss (with ‘strong’ and ‘substantial’ defined as scores at least half a standard deviation above the arithmetical means), depression (defined as a score > 8 on the HADS-D depression scale [12, 13]), significant perceived pain (defined as a score > 3 on the NRS scale), and the occurrence of somatic diseases accompanied by physical pain.

## Results

It has been found that eighty-two caregivers were offspring of the patient; 39 were partners or spouses; and six were friends. A vast majority of caregivers were sole carers and also lived with the patient. Female caregivers slightly outnumbered males (57.5 %). Most offspring and all friends who acted as caregivers had at least a secondary education.

Data on participants’ psychophysical condition and the strength of their relationship to the patient are summarized in Table 1. Participants defined their relationships with patients as strong, and most reported that they had had positive experiences with the patient in the past. Most participants declared a substantial sense of loss and painfulness associated with that loss. Depression was present in 35.4 % of the participants.

Nearly half of caregivers (45.7 %) had had at least one chronic disease diagnosed: 32.3 % had been treated for hypertension, 14.2 % for circulatory insufficiency, 15.7 % for respiratory tract diseases, 20.4 % for diabetes, and 9.4 % took medication for depression.

Just 28 participants (22 %) had diagnoses of pathological states that included pain sensations, whereas 87.4 % reported having pain complaints during the week prior to the study; most often these were backache or headache. The mean intensity of the pain was 4.13 for the group as a whole (*SD* = 4.28; *min* = 0; *max* = 10).

**Fig. 1:**
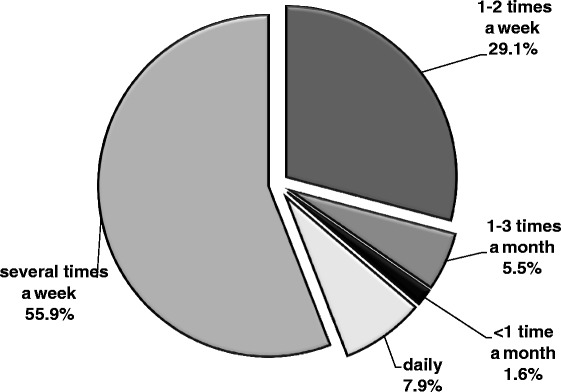
Frequency of taking analgesics without a doctor’s recommendation by caregivers of patients with Alzheimer’s disease

Only one participant had never used analgesics without a doctor’s recommendation (that person had been diagnosed with rheumatoid arthritis, and a daily dose of analgesics was recommended by a doctor). Another participant took analgesics just once or twice a month. The vast majority of participants (93 %) took analgesics without consulting a doctor at least once a week, while 8 % took such medication every day (Fig. 1). Most frequently, participants were using analgesics available without prescription, but 9.4 % of the sample admitted to taking the prescribed drugs more often than directed by the doctor. On average, participants were taking about three doses of analgesics per week and were taking them independently, without consulting a doctor (Table 1).

It was demonstrated that the higher the intensity of physical pain perceived, the higher the assessment of painfulness of loss, and the more acute the sense of loss (Table 2). Moreover, pain correlated positively, albeit weakly, with the strength of emotional relationship and intensity of depression.

**Table 2:** Correlations of factors related to giving care with pain level and use of analgesics

Factors related to giving care	Pain level	Frequency of taking analgesics (without doctor’s recommendation)
Strength of relationship with the patient		
Strength of emotional relationship	0.20*	0.13
Positive experiences	−0.14	−0.09
Sense of loss	0.31***	0.38***
‘Painfulness’ of loss	0.47***	0.51***
Depression	0.19*	0.24**

Note: *n* = 127; *p < 0.05; **p < 0.01; ***p < 0.001

The frequency of taking analgesics without a doctor’s recommendation also correlated positively with painfulness of loss, a sense of loss, and more intense symptoms of depression (Table 2). It is worth noting that the strength of those relationships was greater than that between the same variables and intensity of pain.

The logistic regression analysis indicated that the sense of loss and painfulness of loss were the factors that most profoundly increased the probability of using analgesics without consulting a doctor (Table 3). The painfulness of loss connected with deterioration of the psychophysical condition of a patient with AD increased the chance of taking analgesics nearly four times more than did the sensation of pain itself, or the occurrence of a somatic disease that included pain among its symptoms. Similarly, symptoms of depression nearly doubled the chance of using analgesics without a doctor’s recommendation.

**Table 3:** Probability of using analgesics as a function of caregivers’ psychophysical condition and emotional relationship with the patient

Characteristics of the caregiver	Analgesics (without doctor’s recommendation)		
	OR	95 % CI	p
Strength of relationship with the patient			
Strength of emotional relationship	1.17	0.22, 2.16	0.012
Positive experiences	0.87	0.09, 1.94	0.035
Sense of loss	3.64	1.85, 7.99	<0.001
‘Painfulness’ of loss	4.82	2.03, 8.77	<0.001
Depression	1.86	0.51, 3.21	<0.001
Pain	1.26	0.70, 2.01	<0.001
Somatic pain-related diseases	1.13	0.48, 2.12	0.002

Note: *n* = 127

## Discussion

Our study shows that many caregivers of AD patients report pain complaints and that they frequently take analgesics without consulting a doctor. The sense of loss and painfulness of loss turned out to be the strongest predictors not only of pain intensity but also of using analgesics. This result confirms our hypothesis that pain sensations are relatively intensified and that the use of analgesics is more frequent among this subset of caregivers of AD patients. Conversely, caregivers who did not perceive the loss as emotionally painful also did not experience as much physical pain. Our results therefore also support the concept of social pain [8, 9].

Earlier studies [7] have indicated that people providing care for patients with AD who also have stronger emotional bonds with the patients more often demonstrate somatic, rather than affective, symptoms related to depression. In this study we have demonstrated a positive correlation between depression and intensification of pain. This suggests that the pain reported by participants in our study could also be a symptom of depression. However, the strength of the correlation between depression and pain was low. Moreover, 35.4 % of the participants had significant symptoms of depression, whereas as many as 87.4 % reported the presence of pain. Therefore, not all the pain complaints were necessarily related to the previously occurring depressive or somatic disturbances. Taking into account the fact that most of the pain complaints may be tension-related, we are reminded here of one of the most significant models of caregiving – the stress process model [14]. In accordance with this model, loss of closeness in the caregiver–patient relationship is a secondary stressor, which in turn may negatively influence psychophysical health. Our study indicates that the sense of painfully losing a significant person whom the caregiver has been looking after might constitute a source of social pain, which could lead to the occurrence of pain complaints, by stimulating the anterior cingulate cortex and insula [8, 15–18].

A loss can be perceived as particularly painful when the caregiver lacks support and understanding from others; yet support for caregivers is often substantially limited. This is often a matter of time restrictions, among other factors: caregivers do not have free time to meet other people who could be sources of support (in our study, the caregivers spent an average of 10 h a day providing care). Our participants were often the only person actually providing care to the patient (77.1 %). In the Polish setting, obtaining additional assistance may also be hindered by limited availability of institutional as well as financial support. Another reason for the absence of support may be the limited possibility of honest discussion concerning the difficulties and internal conflicts experienced, for fear of being assessed negatively by others [19, 20]. In Poland, the cultural message that one must sacrifice oneself for other family members is strong. Admitting in public that providing care is tiresome or too difficult, or that the caregiver is unable to cope or already perceives the patient as a stranger, may be met with negative reactions. Frustration connected with lack of understanding, unjust treatment and unfair judgment are other documented sources of social pain [18, 19, 21]. This aspect, however, requires further studies on caregivers dealing with AD patients.

The closeness of the caregiver-patient relationship has emerged as an import predictor of the psychophysical condition of the caregiver. Fauth et al. [22] demonstrated that the sense of being very close and losing this closeness during the provision of care to the patient is associated with more negative self-assessments of physical health. The results of our study supplement those findings by indicating a probable mechanism of origin for this perceived health deterioration: more frequent and profound experiences of pain are an important influence on the general evaluation of one’s health and on the phenomenon of catastrophizing [23, 24]. Catastrophizing is a condition in which an individual perceives her/his ailments as more severe and dangerous, and the possibility of coping with them less probable, than is actually the case. Experiencing unexplained pain – and social pain may be defined as such pain – is conducive to a tendency to catastrophize.

Monin and Schulz [11, 25] state that caregivers’ perceptions of suffering experienced by their loved ones result in increasing distress and negative health consequences in the caregivers themselves. AD patients surely experience frustration and suffering, yet the severity of these experiences may be overestimated by their caregivers, as demonstrated by Schulz et al. [26]. In our research, we did not include the variable ‘perception of patient’s suffering’, yet we expect that it could be part of the aggregated variable we did study, namely the ‘painfulness of loss.’ Apart from that, perceiving the suffering of a loved one may result in fear of losing that person, and may thereby trigger the mechanism of social pain, particularly in people with an anxious/preoccupied attachment style [27].

Also, higher levels of depression are related to lower assessments of health [28]. In our study we observed that depression is connected with a more profound experience of pain. This result is important inasmuch as the study demonstrated a fairly frequent occurrence of intensified depression symptoms (in more than one-third of the participants), whereas only 9.4 % of caregivers were receiving anti-depressive treatment. This speaks volumes about the insufficiently met requirements of support and treatment for caregivers.

Zhu et al. [29] demonstrated that more intense symptoms of depression are connected with an increased probability of taking drugs – both those prescribed by a doctor (OR, 1.112), and those available over the counter (OR, 1.117). In their study, analgesics were not listed by participants as the most frequently used drugs (the list included antihypertensive drugs, drugs used for dyslipidemia, and psychotropic drugs). By contrast, in our study analgesics were the drugs taken most frequently by caregivers. A possible explanation for the discrepancy is that a specialist consultation is relatively difficult to secure, whereas painkillers may be purchased over the counter any time of the day (or night) in close proximity to one’s place of residence [30, 31]. What is more, one can expect analgesics to bring an actual reduction in pain, whether of organic or psychosomatic origin. For example, DeWall et al. [32] found that paracetamol (Acetaminophen) could reduce social pain. It is also worth stressing that over-the-counter painkillers are a class of very cheap drugs that bring relief; one pill costs less than a cigarette. The finding that caregivers use painkillers seems, in this situation, to reflect the reality that such drugs are one of the most readily available strategies for coping with social pain.

In light of the facts mentioned here, it is justified that caregivers should be given not only adequate pharmacological assistance but also support in the form of coping measures for working through the grief they experience while the patient is still alive. This need is especially critical when one considers that the process of losing a person to AD often takes several years. This type of assistance is likely to reduce the psychical suffering of caregivers, but also to minimize their tendency for excessive reliance on analgesics.

### Limitations and advice for future studies

Our study has several limitations. The first is that the group of participants was not representative of caregivers at large. Participants were recruited from Polish caregivers who were town or city dwellers. We should also mention that only 35.5 % of the caregivers invited to participate in the study took part in it. In the future, it would be worthwhile to devote attention to the group of caregivers who refused to participate in the study and who cited lack of time as the reason. One can expect that provision of care by those people is connected with specific bio-social costs. The variable availability of medical procedures and social care in Poland does not allow direct extrapolation of the results to the general population of caregivers of AD patients. It would be worthwhile to repeat the study in different cultural and economic conditions. In future studies, it would also be useful to consider measures of frustration levels associated with the accessibility of medical and social services.

Our study was cross-sectional. The results would be worth verifying in a longitudinal study, to confirm whether the dynamics of the caregiver-patient relationship influence the level of pain complaints reported, and whether these complaints are affected by changes in strategy for coping with stress, including changes related to the use of analgesics (which Zhu et al. [29] observed in relation to other drugs).

Our study was based on single-item thermometer scales, which render the subjective level of sensations experienced by caregivers. The interpretation of results from such measures carries the risk of errors associated with excessive generalization and confounding of concepts. Such factors as, for example, the sense of loss are aggregate variables that may comprise several other constructs. In a future study it would be worthwhile to explore more comprehensively notions of loss and of being close to the patient.

Future studies should also investigate such variables as adequacy of social support, or levels of distress and efficiency of strategies for coping with stress, as potential buffers to the costs of providing care to an AD patient.

## Conclusions

Pain is a symptom commonly experienced by caregivers of AD patients. This phenomenon may be connected to social pain resulting from the experience of losing someone to whom they feel close. Caregivers may resort to excessive use of analgesics as a pain-coping strategy.

## Additional file

Additional file 1:
**Somatic Ailments Questionnaire (originally in polish).** (DOCX 18 kb)

## Abbreviations

AD: Alzheimer’s Disease
HADS: Hospital Anxiety and Depression Scale
NRS: Numerical Rating Scale
