# Supplementary material for: The pain of a heart being broken: pain experience and use of analgesics by caregivers of patients with Alzheimer’s disease
Source: BMC Psychiatry. 2015 Jul 28;15:176. doi: 10.1186/s12888-015-0571-1 (PMC4515928; doi:10.1186/s12888-015-0571-1)
Supplement: Additional file 1: — Somatic Ailments Questionnaire (originally in polish). (DOCX 18 kb) [file 12888_2015_571_MOESM1_ESM.docx]

**Somatic Ailments Questionnaire** *(originally in polish)*

**Have you been suffering from any ailments or chronic illnesses? Which ones?**

…………………………………………………………………………………………………………………………………………………………….

…………………………………………………………………………………………………………………………………………………………….

…………………………………………………………………………………………………………………………………………………………….

**What number best describes your pain on average in the past, including today? Please indicate your answer on the scale below:**

| **0** | **1** | | **2** | | **3** | | **4** | | **5** | **6** | | **7** | | **8** | | **9** | | **10** |
| --- | --- | --- | --- | --- | --- | --- | --- | --- | --- | --- | --- | --- | --- | --- | --- | --- | --- | --- |
| No pain | |  | |  | |  | |  | | |  | |  | |  | | Maximum pain | |

**What has been aching you?** ……………………………..………………………………………………………………………………………………………

**Do you use painkillers?**  No  Yes

**Do you ever use painkillers without a doctor’s recommendation?**  No  Yes

**Which painkillers do you use? How often do you use them? Please fill in below:**

| **Painkiller (name)** | **Dose** | **How many times  per day**  **e.g. 3x /d** | **Frequency of use (especially in the last 30 days)** | **Prescribed by doctor** |
| --- | --- | --- | --- | --- |
|  |  |  |  daily   several times a week   1-2 times a week   1-3 times a month   <1 time a moth |  Yes   No |
|  |  |  |  daily   several times a week   1-2 times a week   1-3 times a month   <1 time a moth |  Yes   No |
|  |  |  |  daily   several times a week   1-2 times a week   1-3 times a month   <1 time a moth |  Yes   No |
|  |  |  |  daily   several times a week   1-2 times a week   1-3 times a month   <1 time a moth |  Yes   No |
|  |  |  |  daily   several times a week   1-2 times a week   1-3 times a month   <1 time a moth |  Yes   No |

**What other medications do you use on a daily basis? Please fill in below:**

| **Medication name** | **Dose** | **Medication use** | **Prescribed by doctor** |
| --- | --- | --- | --- |
|  |  |  Every day   As needed |  Yes   No |
|  |  |  Every day   As needed |  Yes   No |
|  |  |  Every day   As needed |  Yes   No |
|  |  |  Every day   As needed |  Yes   No |
|  |  |  Every day   As needed |  Yes   No |
|  |  |  Every day   As needed |  Yes   No |
|  |  |  Every day   As needed |  Yes   No |

**………………………………………………………………………………….**
